# Watch out, Someone is Watching You!

**DOI:** 10.5005/jp-journals-10071-23160

**Published:** 2019-05

**Authors:** Yatin Mehta, Atul P Kulkarni

**Affiliations:** 1 Medanta Institute of Critical Care and Anesthesiology, Medanta: The Medicity, Gurugram, Haryana, India; 2 Division of Critical Care Medicine, Department of Anesthesiology, Critical Care and Pain, Tata Memorial Hospital, Homi Bhabha National Institute, Mumbai, Maharashtra, India

## Abstract

**How to cite this article:** Mehta Y, Kulkarni AP. Watch out, Someone is Watching You! Indian J Crit Care Med 2019;23(5):205.

The incidence of healthcare-associated infections (HAI) is much higher in the developing countries particularly in India. In one study of International Nosocomial Infections Consortium (INICC),^1^ we found the incidence of central line associated bloodstream infections (CLABSI) to be 5.1/1000 central line days. Ventilator associated pneumonia (VAP) 9.4/1000 mechanical ventilator days and catheter-associated urinary tract infection (CAUTI) 2.1/1000 urinary catheter days, all of which are much higher than the Western incidence. Over and above that we have significant infections with multidrug resistant organisms (MDRO) leading to higher use of expensive antibiotics with increase in length of ICU and hospital stay, mortality and subsequent cost.^2^

Hand hygiene is one of the five key initiatives set out by the World Health Organization (WHO) for patient safety. It recognizes infection control as essential for patient safety and hand hygiene, the most important modality to reduce HAI.^3^ Education, training, hand hygiene and VAP bundle surveillance (conventional visual means), audit and feedback over a time period have shown a significant reduction of VAP from 17.43 /1000 mechanical ventilation days to 10.81/1000, a 38% VAP rate reduction,^4^ in a large multicenter prospective study.

In this issue of IJCCM, there is a study from Jaipur (Video Surveillance of hand hygiene as a tool for monitoring and impact – not an eye wash) which performed video surveillance (VS) and compared it with direct observation (DO) in ICU, HDU, and ER of a tertiary care hospital over a few months and found a significant difference (which is not a surprise!) between the two and VS showed a much lower compliance rate! This was observed, in our opinion, due to the fact that once people realize that someone is watching you, e.g. infection control personnel who are easily identifiable, they will try to be compliant! This is human nature. That is why there are video security cameras installed everywhere!

The compliance monitored by both techniques improved over a few months. This probably happened as the feedback was given to the departments and culprits and as people realize that someone is watching you (VS) they become law abiding/God fearing!

This is a very simple doable process surveillance method which can easily be replicated. Authors have not done statistical analysis between the groups and different months, which would have added to the scientific value of the paper. In the discussion part, the authors mention that employees privacy was maintained but we do not understand how? In fact, we would suggest that after reviewing the VS the noncompliant staff should be told the same day in order to make them realize their mistakes.

Overall it is a simple study with easy reproducibility and practical beneficial effects to reduce HAI. We as intensivists should put in our best effort to improve hand hygiene compliance and contribute to reduction of HAI's and MDROs in our ICUs. In another small study in this issue, the authors conducted a survey (test) on medical students regarding their knowledge of nosocomial infections, hand hygiene, and standard precautions. Seven hundred and twenty-eight MBBS, BDS and BSc students underwent testing pre- and postworkshop on the same. A significant improvement was seen in all parameters posttest. Although it is a very simple study but it does show that education and audit (of knowledge) play important roles in reducing HAI's. Although similar studies have been done in France (developed econonmy)^5^, Ghana^6^ and Ethiopia^7^ (underdeveloped) it is good to see its replication in India! Also catch them young! This is the right time (World Hand Hygiene Week) for this issue to come out.
